# Poly-symptomatology of chronic multi-canalicular benign paroxysmal positional vertigo: a deductive, inductive, and abductive narrative review

**DOI:** 10.3389/fneur.2025.1563295

**Published:** 2025-04-10

**Authors:** Carsten Tjell, Wenche Iglebekk, Peter Borenstein

**Affiliations:** ^1^Norwegian Medical Association, Vennesla, Norway; ^2^Norwegian Physiotherapy Association, Vennesla, Norway; ^3^Swedish Medical Association, Stockholm, Sweden

**Keywords:** vestibular migraine, neck pain, temporomandibular joint pain, cognitive dysfunction, tinnitus, whiplash associated disorders

## Abstract

This narrative review aims to present an overview of the symptomatology of chronic multi-canalicular benign paroxysmal positional vertigo (mc-BPPV) from deductive (what is believed to be known), inductive (what is likely), and abductive (hypothetical) perspectives. The purpose is to recognize these symptoms as expressions of an eventual mc-BPPV when they occur in patients with vestibular migraine, whiplash associated disorders (WAD) and other chronic pain disorders. These symptoms are often considered to be biopsychosocial conditions due to a lack of objective findings, that is, the absence of the findings one is looking for—not the absence of findings generally. The symptomatology of mc-BPPV follows a basic neurophysiologic principle: a disorder in one part of the vestibular system often affects the functions of other parts of the vestibular system. In patients with chronic mc-BPPV, abnormal signals are transmitted as afferents to the vestibular nuclei complex; from there, consistently abnormal efferent reflexes are transmitted. These symptoms can include dizziness, visual disturbances, headache, neck pain, temporomandibular joint region pain, other musculoskeletal pain, involuntary movements, tinnitus, temperature disturbance, and cognitive dysfunction. Therefore, it is necessary to consider the possibility of mc-BPPV in patients with vestibular migraine, WAD and other chronic pain disorders.

## 1 Introduction

The classical dramatic acute clinical presentation of benign paroxysmal positional vertigo (BPPV) is well known; however, among many clinicians, the symptomatology of chronic multi-canalicular benign paroxysmal vertigo (mc-BPPV) is not. The symptoms of chronic mc-BPPV can include dizziness, headache, neck pain, temporomandibular joint (TMJ) region pain, other musculoskeletal pain, involuntary movements, visual disturbances, fatigue, tinnitus, and cognitive dysfunction (1–3).

Patients with chronic mc-BPPV and a history of trauma but few objective findings during standard examination suffer the risk of being diagnosed with a biopsychosocial disorder. It is natural for these patients to be frustrated and discouraged. Many of them are misdiagnosed due to a lack of objective findings, that is, an absence of the objective findings one is looking for—not an overall lack of objective findings. mc-BPPV is common: up to 20% of individuals with BPPV suffer from a multiple canal condition (4, 5). BPPV is considered multi-canalicular if a vertigo/dizziness attack can be evoked in more than one of the standard test positions (6, 7).

According to Ernst et al. (8), any trauma to the head, neck, or craniocervical junction can have a serious impact on the vestibular system. Therefore, previous trauma, for instance, due to traffic accidents, falls, and sports injuries can play a role in chronic mc-BPPV (6, 9–17).

A prospective consecutive cohort observational study was performed with the purpose of recognizing symptoms associated with mc-BPPV (18). The study included 49 patients from 5 physiotherapy clinics. The patients had been referred with the diagnosis of a chronic musculoskeletal disorder (18). The study participants answered a symptom questionnaire and a Dizziness Handicap Inventory (DHI) (19) questionnaire based on an exploratory factor analysis (20). Results showed that 84% of the cohort had a pathological DHI total score. Further, 90% of the patients with a pathological DHI total score had a pathological physical DHI subscale score typical for BPPV (18). Further, a study by Van De Wyngaerde et al. at the Mayo Clinic concluded that a high DHI score and the scores of physical DHI subscale questions in patients suggest a chronic vestibular disorder, such as a chronic mc-BPPV (20).

This narrative review aims to provide an overview of the symptomatology of mc-BPPV from a deductive (what is believed to be known), an inductive (what is likely), and an abductive (hypothetical) perspective. The purpose is to recognize these symptoms as potential manifestations of mc-BPPV when they appear in patients with vestibular migraine, WAD, and other chronic pain disorders.

## 2 Methods

The BPPV diagnostic criteria consensus document from the Committee for the Classification of Vestibular Disorders of the Bárány Society (6) is modeled on the International Classification of Headache Disorders. The document includes both established syndromes (sections 2.1 to 2.4) and emerging and controversial syndromes (sections 3.1 to 3.4). The mc-BPPV syndrome is described in Section 3.3. This classification suggests that mc-BPPV should be considered a condition for which the final consensus has yet to be reached. Thus, we have chosen to define the deductive approach as ‘what is believed to be known’ rather than the definitive ‘what is known.’

This review is based on PubMed database searches. Each symptom is used as a keyword and is combined with the keywords “BPPV” and “benign paroxysmal positional vertigo.”

This tripartite classification has been employed to grade the level of substantiation for the probability that a given symptom can be explained by mc-BPPV. Only symptoms supported by compelling systematic review articles are considered deductive. Original articles that support the hypothesis that a specific symptom may be attributable to mc-BPPV are categorized as ‘most likely,’ i.e., inductive. To our knowledge, this tripartite classification has not previously been applied in similar narrative reviews.

## 3 Epidemiology

According to the BPPV diagnostic criteria consensus document of the Committee for the Classification of Vestibular Disorders of the Bárány Society (6), BPPV is the most common of all vestibular disorders. The cumulative incidence of BPPV during the lifetime in a general population is estimated to be 10% (21). A BPPV illness episode typically lasts from days to weeks before spontaneous remission (22), the frequency of recurrence is roughly 50% (23), and many individuals have several episodes (21, 24). Posterior and horizontal semicircular canal (SCC) involvement is most common in mc-BPPV (6). In contrast, the involvement of the anterior SCC in patients suffering from BPPV is considered rare. In acute mono-canalicular BPPV, the anterior SCC is only diseased in approximately 3% of cases (6, 7, 25–27); however, a considerably higher incidence is observed in multi-canalicular canalolithiasis (3, 11, 28). Moreover, up to 20% of individuals with BPPV suffer from a multiple canal condition (4, 5).

Due to the high recurrence rate, the term “BPPV” can be problematic. How can a disorder be benign when it seriously affects the quality of life (29)? Further, how can a disorder both be paroxysmal and chronic? A more correct term would be “lithiasis”—canalolithiasis for free otoliths or debris—or “cupulolithiasis” for attached lithiasis or debris attached to the cupula. These terms are used by the Bárány Society in their consensus document. However, the term “BPPV” is too well established in the common medical vocabulary to be replaced.

## 4 Pathophysiology in chronic mc-BPPV

BPPV is caused by free-floating otoliths or debris in the SCCs. These otoliths or debris are dislocated from the otolith macula beds in the utricle. The resulting abnormal endolymph flow deflects the cupula (a sensory organ), thus modulating the activity of the vestibular afferents of the affected canal. This increased flow initiates attacks of positional vertigo and nystagmus, a phenomenon known as canalolithiasis (6).

Otoconia that adheres to the cupula of the SCC is known as cupulolithiasis (30, 31). However, cupulolithiasis is not fully accepted as a deductive entity. Bárány Society (6) rubricates the condition cupulolithiasis of the posterior SCC among emerging and controversial syndromes (Section 3.2). Positional nystagmus is observed in the vast majority of healthy individuals (32, 33). Asymptomatic positional nystagmus could be the result of compensated cupulolithiasis. Individuals with cupulolithiasis as well as vestibular neuritis, that is, static balance disorders, are asymptomatic (in line with the principles of neural plasticity) (34, 35). Dynamic balance disorders such as BPPV (canalolithiasis) do not improve through compensating exercises because such disorders are characterized by free-floating otoliths and debris transmitting varying abnormal signals from time to time in response to the same stimuli (35).

The literature has paid scant attention to the anterior SCC because the latter is described as rare (6, 7, 25–27) but is not uncommon in mc-BPPV (3, 11, 28). In the upright position, the cupula of the anterior SCC is in the horizontal plane. Even minute movements of the head, for instance, due to eating, talking, and walking will have an influence on the cupula because of the free-floating otoliths that can be present on top of the cupula (4). Additionally, many studies have documented a high degree of chronic morbidity in mc-BPPV patients, when the anterior SCC is involved (3, 11, 28, 36, 37).

Practicing Brandt–Daroff exercises for the treatment of an acute posterior BPPV can result in involvement of the anterior SCC; these exercises are a sequence of rapid lateral head/trunk tilts from side-to-side, repeated in series to promote dispersion of the debris toward the utricular cavity (38). Patients diagnosed with anterior SCC involvement often report having previously practiced the Brandt–Daroff exercises for the treatment of an acute posterior BPPV. During these exercises, debris reaches the crus commune—the common part of the posterior and the anterior SCC—on the way to the utricle. However, on its return, the debris can easily end up in the anterior SCC (39, 40). This idea is supported by Vannucchi et al. (41), who said: “Moreover, atypical positional nystagmus can sometimes be transformed into paradigmatic benign paroxysmal positional nystagmus, simply by means of diagnostic or therapeutic maneuvers. The canal clot in fact could move into the *SCC*s by the effect of the changing gravity vector.” Therefore, we raise the question of whether involvement of the anterior SCC can be iatrogenic in some cases and if Semont repositioning can carry the same risk (42).

**Figure 1:**
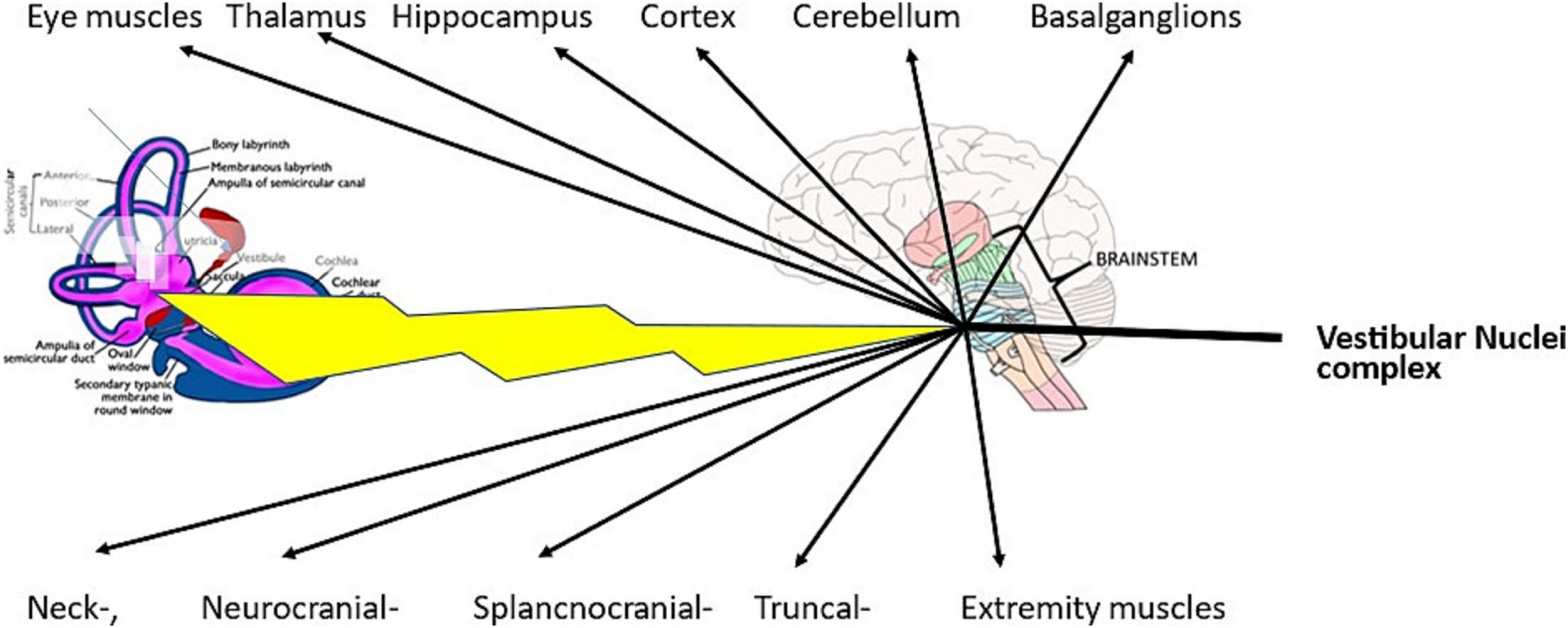
In mc-BPPV, abnormal signals are transmitted from a diseased labyrinth to the healthy functioning vestibular nuclei complex. From there, the abnormal signals are transmitted directly via the vestibulo-spinal reflexes or different cranial nerve nuclei to their end organs.

In peripheral vestibular disorders, the vestibular nuclear complex in the brainstem is healthy and, therefore, functions consistently. In patients with BPPV and in patients with chronic mc-BPPV, abnormal signals are transmitted as afferents to the vestibular nuclei complex; from there, consistently abnormal efferent reflexes originate. These reflexes are the well-known vestibulo-ocular reflexes and the vestibulo-spinal reflexes (Figure 1). Moreover, there are several other active vestibular reflexes that can lead to symptoms that are usually not attributed to a vestibular disorder (43, 44).

Vestibulo-thalamo-cortical activity, posited in the vestibular migraine review articles by Espinosa-Sanchez and Lopez-Escamez (45) and by O’Connell et al. (46), is just one of several reflexes originating in the vestibular nuclei complex. Further, the vestibulo-thalamo-cortical reflex is probably one cause of cognitive difficulties (47). All the symptoms shown in Table 1 can be explained by the above-mentioned vestibular connections.

**Table 1:** Symptom frequencies (%) of three cohorts of patients suffering from mc-BPPV and one cohort of patients with chronic musculoskeletal disorders.

	mc-BPPV (3) *N* = 163 (%)	mc-BPPV (1) *N* = 69 (%)	mc-BPPV (2) *N* = 39 (%)	Musculo-skeletal (18) *N* = 41 (%)
DHI score	-	55	48	45
Isolated rotatory vertigo	8	20	5	12
Combined rotatory and nautical vertigo/dizziness	92	81	95	51
Nausea	74	74	72	39
Headache	82	75	95	98
Neck pain	82	87	92	93
Generalized pain	13	40	54	53
Involuntary movements	35	-	36	-
Impaired short-term memory	67	65	82	61
Concentration problems	72	81	87	81
TMJ region pain	50	-	56	-
Tinnitus	44	54	49	37
Fatigue	88	85	97	83
Sensation of globus	35	48	-	-
Temperature disturbance	61	51	62	-

The references (1–3, 18) refer to articles in which the symptom frequencies are published.

## 5 Symptoms of patients with chronic mc-BPPV

The clinical presentation of mc-BPPV is multifaceted. Table 1 shows the frequencies of different symptoms in three patient cohorts with mc-BPPV and one patient cohort with chronic musculoskeletal disorders. The authors (1–3) find it likely that these symptoms are caused by mc-BPPV because the symptoms can ameliorate immediately after repositioning therapy. A similar argument has been used by the Bárány Society criteria of BPPV (4) as well as in the American Clinical Practice guideline (7) to support this diagnosis: “If positional nystagmus disappears immediately after positional therapy, this strongly supports the diagnosis of BPPV.”

In a retrospective consecutive cohort of 163 patients with chronic mc-BPPV and a history of trauma (3), 98% of the patients fulfilled the Bárány Society criteria of a probable vestibular migraine (48) and 63% fulfilled the Fukuda criteria of myalgic encephalomyelitis/chronic fatigue syndrome (ME/CFS) (49). Further, 100% of the patients diagnosed with whiplash-associated disorders (WAD) (50) suffered from chronic mc-BPPV. These findings support the idea that chronic mc-BPPV can trigger symptoms of vestibular migraine, ME/CFS, and WAD. Similar symptom frequencies were found in two other cohorts of patients with chronic mc-BPPV (1, 2) (Table 1).

## 6 Discussion of symptoms

In epidemiology, there is always the question of coincidence versus coexistence. We argue in favor of the latter by following a basic neurophysiologic principle: a disorder in one part of the vestibular system often affects the function of other parts of the vestibular system (43).

### 6.1 Vertigo and dizziness: deductive issues

The central nervous system (CNS) receives afferent information from the vestibular, visual, and sensorimotor systems. These signals converge in multiple areas and are important for general equilibrium, body orientation, and oculomotor control. Abnormal afferent input from one of these systems can result in a mismatch. Under-and overactivity in afferent input can result in abnormal postural control. The resulting mismatch due to conflicting afferent information is assumed to be the cause of symptoms such as dizziness and unsteadiness (38).

As Table 1 shows, isolated rotatory vertigo (i.e., a typical acute BPPV attack) without any additional dizziness episodes is infrequent among patients with chronic mc-BPPV. According to criteria 3.3 of Bárány Society (6), besides attacks of positional vertigo, patients may have prolonged mild unsteadiness. Occasionally, patients may complain of positional dizziness, unsteadiness, and vegetative symptoms such as nausea, sweating, and tachycardia (51).

Large population-based studies indicate that dizziness (including vertigo) affects approximately 15% to over 20% of adults yearly. Vestibular vertigo accounts for about a quarter of dizziness complaints and has a 12-month prevalence of 5% and an annual incidence of 1.4% (52). Studies have documented the high prevalence of BPPV and vestibular migraine and comorbid anxiety across the population. Neuhauser (52) pointed out that BPPV and vestibular migraine are largely underdiagnosed, while Menière’s disease, which occurs roughly 10 times less frequently than BPPV, appears to be over diagnosed (52).

Dizziness prevalence rises with age and is approximately two to three times higher in women than in men. Imbalance has been increasingly studied as a highly prevalent complaint particularly affecting healthy aging (52). The recent World Falls Guidelines recommend formal assessment for BPPV in older adults at risk of falling, but only if they report vertigo. This recommendation ignores the data pointing out that many older adults with BPPV experience dizziness as vague unsteadiness rather than as vertigo (53).

Dizziness is a frequent complaint following head trauma. Among patients who suffer from concussion (mild traumatic brain injury; mTBI), dizziness is second only to headache in frequency. Among the main diagnoses regarded as causes of post-concussive dizziness are central vestibular disorders and BPPV (54).

Vertigo/dizziness has become part of daily life among many patients with a long history of disease. This situation is evidenced in a study of a group of patients suffering from a chronic musculoskeletal disorder (18), which reported that 84% of the cohort had a pathological DHI total score. Ninety percent of the patients with a pathological DHI total score had a pathological physical DHI subscale score typical for BPPV (18). In the same cohort, less than two-thirds presented any balance-related symptoms.

This normalization of symptoms is illustrated by a boy with chronic headache and chronic mc-BPPV, who asked, “Isn’t it normal to be dizzy when playing soccer?” Unfortunately, many such patients are misdiagnosed because attention is focused on the dominant symptom.

Another example is a 50-year-old man who had been a victim of a road traffic accident. His main symptom was severe headache. He visited several specialists and always reported “the dizziness came first.” Since headache was his dominant symptom, no interest was paid to the dizziness. After being diagnosed with chronic mc-BPPV and receiving otolith repositioning, the headache ceased.

Dizziness frequently occurs in shopping centers, and a visit to a shopping center is often followed by increased intensity of other symptoms. When moving in narrow indoor streets, the peripheral visual field is substantially stimulated, activating the optokinetic system that directly communicates with the vestibular nuclei complex (43). In addition to the optokinetic signals, the vestibular nuclei complex receives disturbing vestibular signals due to frequent turning of the head and eyes toward new objects of interest (43). Frequent turning of the head is tolerated by a healthy person, but not by one suffering from mc-BPPV and having dislodged otoliths. Therefore, a visit to a warehouse can be challenging given the combination of many different postural stimuli that can lead to a postural mismatch.

### 6.2 Vestibular migraine: a deductive and inductive perspective

A vestibular migraine is thought to result from vestibulo-thalamo-cortical activity (45, 46). This activity originates from the vestibular nuclei complex. The mechanism of vestibular migraine is elaborated in the diagnostic criteria of vestibular migraine by the International Bárány Society (48). These criteria were updated in 2021 (55).

Migraine attacks can be induced by vestibular stimulation. For instance, caloric stimulation triggers a migraine attack within 24 h in patients with migraine (56). This observation aligns with the theory that mc-BPPV can trigger vestibular migraine (3).

The vestibular symptoms used to diagnose vestibular migraine include (51, 55):

- Spontaneous vertigo, compassing internal vertigo with a false sensation of self-motion
- External vertigo with a false sensation that the visual surroundings are spinning or flowing
- Position vertigo occurring after a change in head position
- Visually-induced vertigo triggered by a complex or large, moving visual stimulus
- Head-motion-induced vertigo occurring during head-motion
- Head-motion-induced dizziness with nausea.

Dizziness is characterized by a sensation of disturbed spatial orientation. The vestibular symptoms mentioned above are common in mc-BPPV.

The duration of episodes of vestibular migraine or mc-BPPV is highly variable. Approximately 30% of patients with vestibular migraine have episodes lasting minutes, 30% have episodes lasting hours, and another 30% have attacks lasting several days; the remaining 10% have attacks lasting only seconds. These episodes are related to head motion, head position, and visual stimulation (57). Associated symptoms vary from time to time and may differ between episodes. Visual auras are among the most commonly associated symptoms, and these auras are characterized by bright scintillating lights or zig-zag lines, often with a scotoma that interferes with reading (46).

The complexity of vestibular disorders is demonstrated by the finding that migraine is more common among patients with Menière’s disease than among healthy controls (56). Patients presenting with symptoms of both vestibular migraine and Menière’s disease have frequently been reported. Migraine headaches, photophobia, and even migraine aura are frequent during Menière’s disease attacks (58, 59). However, the pathophysiologic relationship between these features remains uncertain (60, 61). According to Dlugaiczyk et al. (62) the phenomenon of “recurrent vestibular symptoms not otherwise specified” most likely represents symptoms on a spectrum of mild or incomplete variants of known vestibular disorders.

### 6.3 Myotatic spinal reflexes and the development of musculoskeletal pain: a deductive and inductive perspective

The human body, which has a high center of gravity with many adjustable segments on top of each other and a very small supporting base, is maladjusted for vertical balance. The advantage of this multisegmented construction is that it allows the equilibrium to be maintained in many different positions, including when moving (63). The resulting amount of information has to be processed is a challenge for the brain. It is, therefore, easy to see why some people suffer from balance disorders; it is, conversely, remarkable that most people do not.

Postural control is mainly consolidated through efferent vestibulo-spinal (43) and vestibulo-reticular reflexes (43, 64). From a teleological perspective, these reflexes aim to prevent an individual from falling. The lateral vestibulo-spinal reflex is involved in the control of muscle tension in the torso and extremities. Further, the medial reflex, also called the vestibulo-collic reflex, is involved in the control of the neck (65). Moreover, the reticular formation functions to maintain a level of tonus and integrate information from several neural centers. On the afferent side, the vestibular nucleus complex receives the cervicocollic reflex as well as the weaker cervicoocular reflex (43).

A disturbance in the delicate balance of facilitatory and inhibitory signals in the myotatic spinal reflexes is the reason behind the development of musculoskeletal pain (44, 64). The most common way to compensate for a dynamic balance disorder appears to be the static use of antigravity muscles (66). However, it is well documented that static use generates an accumulation of pain-generating substances such as arachnoid acid, bradykinin, and histamine (67–69). Consequently, among patients with chronic mc-BPPV, neck pain is a common symptom with a prevalence of 80–90% (1–3).

Based on the published literature, the Bárány Society Classification Oversight Committee (70) takes the view that there is no evidence supporting a mechanistic link between an illusory sensation of self-motion (i.e., vertigo—spinning or otherwise) and neck pathology and/or symptoms of neck pain—either by affecting the cervical vertebrae, soft tissue structures, or cervical nerve roots. When a combined head and neck movement triggers an illusory sensation of spinning, there is either an underlying common vestibular condition such as migraine or BPPV.

During static postures in screen work, the eyes move. The smooth pursuit eye movement system involves the vestibular nuclear complex. Visual signals travel via the inferior olive and activate the Purkinje cells in the midcerebellum. The midcerebellum areas also receive afferent vestibular signals, among other peripheral vestibular signals from lithiasis-affected SCCs and signals from the afferent cervicocollic reflex. Outflow from cerebellar Purkinje cells terminates at secondary excitatory and inhibitory vestibular neurons, thus affecting the vestibulo-ocular reflex and the vestibulo-spinal reflexes (43).

Symptoms such as visual disturbances, concentration impairment, and muscle tension are common in patients with chronic mc-BPPV. Screen work, therefore, increases the intensity of these symptoms. In a prospective consecutive cohort study of patients with chronic mc-BPPV at 7-month follow-up after otolith repositioning, 75% of the patients had experienced a reduction in neck pain (2).

### 6.4 Involuntary movements: abductive perspectives

In two studies (2, 3), one-third of patients with chronic mc-BPPV reported experiencing involuntary movements. During otolith repositioning maneuvers, the authors observed three types of movements, namely, tremor-like, athetotic-like, and hemibalistic-like. Furthermore, nearly two-thirds of the patients in a treatment study (2) had a substantial improvement in involuntary movements after undergoing otolith repositioning. It is likely that these involuntary movements can be explained by abnormal vestibular activity that creates a mismatch in the signals to and from the vestibular nuclear complex.

According to Stiles and Smith (71), involuntary movements in vestibular disorders can be explained through an unspecified “vestibulo-basal ganglia” reflex. The dorsolateral striatum is likely the main input area for vestibular signals in the basal ganglia that have an influence on motor control. Houser et al. (72) documented a series of 26 patients with paroxysmal kinesigenic choreoathetosis, that is, the patients’ symptoms were precipitated by sudden movement. Moreover, Chew et al. (73) reported the triggering of attacks by vestibular stimulation in a few cases of idiopathic paroxysmal kinesigenic choreoathetosis.

### 6.5 Cognitive difficulties: inductive perspectives

As shown in Table 1, 60–80% of patients suffering from chronic mc-BPPV have occurrence of short-term memory and concentration difficulties. Abnormal signals known as the vestibulo-thalamo-cortical reflex are transmitted from the vestibular nuclei complex. This reflex is important in the development of cognitive dysfunction (47). In a treatment study (2), 80% of patients with chronic mc-BPPV had an improvement in their concentration difficulties after otolith repositioning.

The findings by Bigelow et al. (47) indicate that vestibular impairment is associated with an increased risk of, among others, cognitive comorbidity. The vestibular system is widely connected, anatomically, to regions of the cerebral cortex, hippocampus, and amygdala. Abnormal or missing vestibular inputs may lead to the impairment of these cognitive and affective circuits; however, further longitudinal research is required to determine whether these associations are causal (47). Additionally, the findings of a comprehensive review indicate that vestibular disorders can result in impairments across various aspects of cognitive functioning (74).

Cognitive impairment can also be a predictor of poor prognosis after whiplash trauma (75). Thirty-two individuals from a cohort of 163 patients suffering from chronic mc-BPPV fulfilled the European criteria for WAD III (50); all the 32 individuals also fulfilled the Bárány Society criteria (point 3.3) for mc-BPPV (3).

### 6.6 TMJ regional pain and tinnitus: an inductive perspective

Cervical biomechanical disturbances can affect the TMJ region, the face, and the cranial area. There are various causes of TMJ region pain. In patients with WAD, there can be radiological demonstrable pathology in the TMJ joint (76, 77).

As shown in Table 1 (two studies), TMJ region pain is present in about half of patients with chronic mc-BPPV. Moreover, TMJ region pain that is not caused by pathoanatomical changes can be explained by abnormal activity in the trigeminus, facial, and glossopharyngeal nerves and is triggered by lithiasis-induced vestibular signals (78). The motor root of the trigeminal nerve innervates mastication muscles the masseter, temporalis, and pterygoids muscles besides a few muscles of the palate (tensor veli palatine), middle ear (tensor tympani), and upper neck (mylohyoid and anterior belly of the digastric muscle) (78).

An investigation of 163 patients with chronic mc-BPPV indicates a high incidence of involvement of the anterior SCC (3). This finding corresponds to an earlier study that documented the relief of TMJ pain in 13 out of 19 patients after treatment of the anterior SCC with a forward-somersault maneuver (2).

Associations of tinnitus with signs and symptoms of TMJ disorders have been reported in several studies (79–82). It has been documented that patients suffering from tinnitus and TMJ disorders are most often young females with better hearing function than patients with tinnitus without TMJ symptoms (82).

In cases of intermittent tinnitus, the non-classical auditory pathways are abnormally activated by vestibular input (83, 84). The non-classical ascending auditory pathways receive input from other sensory systems such as the vestibular, visual, and somatosensory systems. The perception and intensity of tinnitus can, in some individuals, be somatically modulated. Somatosensory modulation of tinnitus has its origins in the complex somatosensory–auditory interactions that are evoked from musculoskeletal anatomic regions such as muscles related to the TMJ, craniocervical junction, cervical vertebrae, neck, and shoulders (85–88).

According to Shore et al. (89), afferent somatosensory information from the periphery to the secondary sensory neurons in the brainstem—specifically the spinal trigeminal nucleus and dorsal column nuclei—are connected. The cochlear nucleus receives excitatory projections from these structures, which are associated with proprioceptive and cutaneous sensations. Auditory brainstem-response recordings in the dorsal cochlear nucleus indicate that these pathways are physiologically active. Similarly, activation of the trigeminal ganglion causes excitation in some dorsal cochlear nucleus units and inhibition in others. The modulation of firing rate and synchrony in dorsal cochlear nucleus neurons by somatosensory input is the physiological basis of somatic tinnitus, distinct from the classical continuing-treble or high-frequency tinnitus.

### 6.7 Glossopharyngeal nerve-related symptom: abductive perspectives

Given the lack of confirmatory studies, this section relies only on personal experiences. Hence, we must call this section abductive. The globus phenomenon is a non-painful sensation of a lump or a foreign body in the throat, frequently improving with eating before returning half an hour later. Although globus is a common symptom (Table 1), its etiology is poorly understood and controversial. In recent years, the focus on globus has been on somatic causes in contrast to the past when globus was labeled as a hysterical symptom (90, 91). The symptom of globus can be explained through triggered glossopharyngeal nerve activity.

### 6.8 Temperature disturbance: inductive perspectives

Temperature disturbance is a rather common symptom among individuals with mc-BPPV. In a cohort of 39 patients experiencing chronic mc-BPPV, 26 patients reported temperature disturbance as a symptom. At follow-up 7 months later, 63% of patients reported improvement after otolith repositioning (2).

According to the Barany Society complaints during the attacks include among others unsteadiness and vegetative symptoms such as nausea, sweating, and tachycardia (6).

Temperature disturbance can also be explained by a vestibulo-thalamic reflex disturbance mediated by interneurons between the thalamus and hippocampus (43). This assumption is supported by the following observation: The active system for temperature regulation consists of the regulatory pathways and effector organs that elevate skin blood flow and sweat rate. Physiological regulation of cutaneous vasodilation and sweat production is based on the communication of thermal afferents to the CNS (92). Brain metabolism during whole-body hyperthermia (deep body temperature of 38.6°C) was demonstrated through quantifying changes in positron emission tomography imaging. Among other regions, the hypothalamus and thalamus showed increased metabolism that seems to be critical (93). Other data support the existence of the complex and the extensive influence of the neurovestibular system on the homeostatic, circadian, and possibly autonomic temperature-regulatory systems (94).

### 6.9 Fatigue: abductive perspectives

In a cohort of 163 patients with chronic mc-BPPV, 80% suffered from fatigue. Sixty-three percent of the patients fulfilled the Fukuda criteria for ME/CFS (49). The study concluded that mc-BPPV can trigger ME/CFS (3). Further, in a study in which 39 patients with chronic mc-BPPV were followed up 7 months after undergoing otolith repositioning, 84% of them reported improvements; this result also correlates with an improvement of the degree of vertigo experienced by the patients (2).

Several possible explanations for ME/CFS are presented in a narrative review by Row et al. (95), including a neuroendocrine abnormality. A similarity in symptoms between ME/CFS and adrenal insufficiency has prompted an investigation into abnormal hypothalamic–pituitary–adrenal axis function. Statistically lower cortisol levels have been identified in patients with ME/CFS compared with conditions among healthy controls. Whether these findings are primary or secondary to the ME/CFS condition is unknown. However, these findings could be a consequence of a vestibulo-thalamo-hypothalamic reflex activity.

Chronic pain management, changes in posture due to decreased balance, and cognitive difficulties are energy-demanding.

### 6.10 Meniere-like appearances of chronic mc-BPPV: inductive and abductive perspectives

Physio-mathematical models show that the pathophysiology of BPPV is based on the prerequisite that in BPPV, a certain concentration of otoconia reaches a “critical mass” in the affected SCC. Agglomeration of these particles influences the hydrodynamic effect of otoconia moving in the canal (96, 97). Resolution of BPPV may occur due to a dilution of the “critical mass” of otoconia. As with Menière’s disease, the cause of remission might be related to endolymph density. Even though the level of total protein in the endolymph is low, specific proteins play a role in the protection of cell membranes in the membranous labyrinth. A small amount of glycosylated endolymph proteins is continuously secernated; these proteins are primarily required to maintain the structural and functional entirety of the tectorial membrane, the otoconia-complex membrane, and the cupula. These proteins are complex macromolecules and cannot be eliminated transversely through the peripheral compartment but are instead eliminated longitudinally through the endolymphatic sac. A dysfunctional endolymphatic sac impedes the clearance of these macromolecules, contributing to a chemical imbalance in endolymph and endolymphatic hydrops that probably results in a change in viscosity (98). Additionally, it should be mentioned that 1,211 proteins in the endolymphatic sac have been detected per-operatively in neurinoma patients (99).

There is a recognized association between Menière’s disease and BPPV. BPPV comorbidity with Menière’s disease is mostly observed in individuals with an ear affected by hydrops, females, and patients with more advanced disease. A study found that in patients with both BPPV and Menière’s disease, recurrence was more likely and needed more canal repositioning maneuvers than in patients without Menière’s disease (100). Moreover, Hornibrook and Bird (101) hypothesized that the fundamental cause of Menière’s disease might be detached saccular otoconia. Their hypothesis is based on animal studies where hydrops in the cochlea, saccule, and utricle were caused by a blockage of the ductus reuniens (from the cochlea to sacculus) and the endolymphatic duct. Additionally, cone beam computed tomography images have shown apparent obstructions of the ductus reuniens, saccular duct, and endolymphatic sac (101).

Many patients with chronic mc-BPPV have symptoms with similarities to endolymphatic hydrops: they experience exacerbations from upper airway infections, prolonged stress, air travel, or periods with low atmospheric pressure. This outcome aligns with the triggers of Menière’s spells (102–104). Moreover, a recurrence or exacerbation after air flight can be avoided by a prophylactic intake of 30 mg of furosemide—the same dosage used as prophylaxis among patients with Menière’s disease. However, a Cochrane report has given limited support for the efficacy of diuretics in this context. Diuretics are generally used as first-line therapy for Menière’s disease, but studies that support using diuretics provide scant evidence (105). Some women suffer from chronic mc-BPPV exacerbations that are related to menstruation, and these women achieve better management of their disease after receiving a hormonal therapeutical intervention (106, 107).

Luryi et al. (108) reported that factors such as a history of Menière’s disease and trauma have no influence on the recurrence of BPPV episodes. Instead, they identified female gender and a history of previous BPPV as the only factors of relevance. It is evident that discussions around vestibular disorders are ongoing, as reflected in the following statement by Strupp et al. (109): “There is still a great need for state-of-the-art randomized controlled treatment trials in most peripheral vestibular disorders.”

## 7 Conclusion

According to the Austrian British philosopher Popper, one cannot prove anything—one can only disprove. In this narrative review, vertigo, dizziness, headache, and neck pain are deductively considered to be related to mc-BPPV. Symptoms such as muscular pain, cognitive dysfunctions, TMJ region pain, tinnitus, and temperature disturbances can be inductively considered to be related to mc-BPPV. Finally, the involuntary movements, the globus phenomenon and fatigue are hypothetically related to mc-BPPV.
